# Physical characteristics and antimicrobial properties of *Apis mellifera*, *Frieseomelitta nigra* and *Melipona favosa* bee honeys from apiaries in Trinidad and Tobago

**DOI:** 10.1186/s12906-020-2829-5

**Published:** 2020-03-17

**Authors:** Elijah Brown, Michel O’Brien, Karla Georges, Sharianne Suepaul

**Affiliations:** Department of Basic Veterinary Sciences, School of Veterinary Medicine, Faculty of Medical Sciences, The University of the West Indies, St Augustine Campus, Building #49 Eric Williams Medical Sciences Complex, Mt Hope, Trinidad, West Indies Trinidad and Tobago

**Keywords:** Honey, Trinidad and Tobago, Stingless bees, Natural antimicrobials, Apitherapy

## Abstract

**Background:**

Honey is a versatile and complex substance consisting of bioactive chemicals which vary according to many bee and environmental factors. The aim of this study was to assess the physical and antimicrobial properties of five honey samples obtained from three species of bees; two stingless bees, *Frieseomelitta nigra* and *Melipona favosa* and one stinging bee, *Apis mellifera* (fresh and aged honey). Samples were acquired from apiaries across Trinidad and Tobago. An artificial honey, made from sugar, was also used for comparison.

**Methods:**

Physical properties such as appearance, pH, moisture content, sugar content and specific gravity were determined. Antimicrobial activity was assessed utilizing the agar diffusion assay and comparison to a phenol equivalence. The broth microdilution test was performed to determine the minimum inhibitory concentrations (MICs) and the minimum bactericidal concentrations (MBCs) of the five honey samples against four common pathogens, including *Staphylococcus aureus*, *Escherichia coli*, *Streptococcus pyogenes* and *Haemophilus influenzae*.

**Results:**

All honey samples were acidic, with pH values ranging from 2.88 (*M. favosa* of Tobago) to 3.91 (fresh *A. mellifera*). Sugar content ranged from 66.0 to 81.6% with the highest values detected in stinging bee honeys of the *A. mellifera* (81.6 and 80.5°Bx). Moisture content ranged from 16.9% for aged *A. mellifera* honey (from Trinidad) to 32.4% for *F. nigra* honey (from Tobago). The MICs (2 to 16%) and MBCs (2 to 32%) of stingless bee honeys were lower than that of stinging bee and artificial honeys (16 to > 32%). Stingless bee honeys also exhibited a broad spectrum of antimicrobial activity against both Gram-positive and Gram-negative organisms with higher phenol equivalence values (4.5 to 28.6%) than the *A. mellifera* honeys (0 to 3.4%) against the isolates tested. *M. favosa* honey of Tobago displayed the greatest antimicrobial activity as indicated by the high phenol equivalence and low MIC and MBC values.

**Conclusions:**

Stingless bee honeys from Tobago showed the greatest antimicrobial activity when compared to the other honeys used in this study. *M. favosa* honey of Tobago showed the most potential for use as medicinal honey.

## Background

For millennia, the medicinal properties of honey have been used by mankind. This popular bee product is versatile and is effective against microbes [1–4], reducing inflammation [5], combating cancer [6], hypertension [7], and liver disease [8], aids in wound healing [3, 9] and provides cough relief [10]. An important therapeutic quality of honey is its antimicrobial activity [11–14]. Investigations into alternative, natural antimicrobial agents such as honey is of immense importance to safeguard human and animal health.

Honey displays many different antimicrobial properties, some of these have been elucidated by Kwakman et al. (2010) [15]. These include a high osmolarity, low pH, methylglyoxal (MGO), hydrogen peroxide and bee defensin-1 (a peptide with antimicrobial properties) [15]. High concentrations of sugars such as glucose, fructose, sucrose and maltose, combined with a low water content in honey causes osmotic stress to microorganisms [16–18]. Acids such as gluconic, phenolic and amino acids contribute to the low pH of honey and are also responsible for the unique taste of the various types of honey [19]. MGO is formed in two ways, through the prolonged storage of carbohydrate containing substances and by the conversion of dihydroxyacetone (DHA) present in honey [20]. Enzymes such as glucose oxidase present in bees aids in the conversion of glucose to gluconic acid and hydrogen peroxide [21]. Bee defensin-1 or royalisin is a peptide secreted by the hypopharyngeal gland of the honey bee [15]. It protects the bee against American Foulbrood and has potent antimicrobial activity [15, 22]. Although honey may contain these compounds and enzymes, there is immense variability in the antimicrobial activities of honeys of different origins due to factors such as the species of bee [12, 14], geographical location [23], soil type [24], floral source [25], season [26] and age of honey [11, 27].

Manuka honey is one of the most popular and extensively researched medicinal honeys [1, 28–31]. It is produced in Australia and New Zealand from a monofloral source known as the Manuka tree (*Leptospermum scoparium*) [28]. Some commercial preparations containing this medicinal grade honey have been developed and are marketed globally, inclusive of L-Mesitran® ointment produced by Aspen Medical and Medihoney® produced by Derma Sciences. These products have been approved by the Food and Drug Administration (FDA) of the USA. The antimicrobial properties of indigenous honeys from Chile [31], Saudi Arabia [32], Poland [14] and Algeria [23] have been published but there is no published data on the medicinal properties of honey from Trinidad and Tobago.

Although, some imported commercial preparations are available in Trinidad, most citizens tend to utilize honeys that are locally produced. These honeys have not been scientifically tested and only anecdotal accounts of their efficacy exist. Hence, it is essential to study local honeys in Trinidad and Tobago of different bee origins to elucidate the variations which may exist in their antimicrobial efficacies.

Trinidad and Tobago is the southernmost island of the Caribbean which lies at 10.69°N, 61.22°W and experiences a wet and dry season. About 5% of the country is covered by tropical rainforests, which provide a wide range of floral sources for bees. Honey in Trinidad and Tobago is produced by both stinging and stingless bees, however the honey of the stinging bee (known as *Apis mellifera*) is more widely consumed and preferred because of its greater yield and sweet taste.

Historically, it is thought that the stinging bee species *Apis mellifera*, was bought to Trinidad by European colonizers in 1498. This Italian subspecies of honey bees colonized Trinidad until the Africanized subspecies of *Apis mellifera*, was introduced to the country in 1979. The Africanized honey bees proved to be better adapted to life on the islands than the previous Italian honey bees and eventually only the Africanized subspecies could be found [33, 34].

There are also eight main species of stingless bees (Meliponini) which are indigenous to Trinidad and Tobago. These include *(Trigona) Frieseomelitta nigra* (Petit Angel), *Trigona amalthea* (Pegone), *Partamona nigrior* (Caca Pegone), *Nannotrigona testaceicornis* (Irai), *Melipona favosa* (Eric/Moca Chiquita), *Melipona trinitatis* (Guanot/Moca Grande), *Lestrimelitta limao* (Limon kaab) and *Plebeia tobagoensis* [35, 36].

This study aimed to determine the physical properties (inclusive of pH, moisture content, sugar content and specific gravity) of five honey samples (two *Apis mellifera*, one *Melipona favosa* and two *Frieseomelitta nigra*) obtained from apiaries across Trinidad and Tobago. We evaluated the minimum inhibitory concentrations (MICs), minimum bactericidal concentrations (MBCs) and phenol equivalences of these honeys against common bacteria isolates of *Staphylococcus aureus*, *Escherichia coli*, *Streptococcus pyogenes* and *Haemophilus influenzae*.

## Methods

### Honey samples

Honey samples were obtained from local apiaries during January to June (dry season) of 2016. These samples were available at the time of visit to the apiaries. Samples were classified as fresh if tested within one month of harvest and aged if tested one year after harvest. A total of five samples were obtained, two *Apis mellifera* samples from Trinidad (one was fresh and the other was aged), two *Frieseomelitta nigra* samples (both were fresh, one from Trinidad and the other from Tobago) and one fresh *Melipona favosa* honey sample from Tobago.

In addition, an artificial honey was produced in vitro according to specifications by Cooper et al. (2002) containing 40.5% fructose, 33.5% glucose, 7.5% maltose, 1.5% sucrose in 17 mL of sterile distilled water [37], which was used as a control. All honeys were placed into 10 mL sterile sealable glass vials and stored in the dark at room temperature (24 °C) until tested. To determine the presence of bacterial or fungal contaminant, blood agar plates (BAPs) and Sabouraud Dextrose Agar (SDA) plates were inoculated with 10 μl of each honey. The inoculated BAPs were incubated at 35 °C for 24 h and the SDA plates were incubated at 30 °C for 3–7 days, after which these plates were observed for any fungal or bacterial growth.

### Physical honey properties

The colour of the honey was observed and recorded and the pH of each honey sample was determined using a pH meter (Mettler Toledo Seven Easy pH meter In Lab 413). A honey refractometer (GX Pro New Design 58–90% Brix Honey Refractometer RHBM-90ATC) was used to determine the water and sugar content of each sample and the specific gravity of the honey was calculated using the following equation [38]:

$$ Specific\ Gravity=\frac{145}{145-\mathrm{degrees}\ \mathrm{Baum}\acute {\mathrm{e}}} $$

### Bacterial isolates

Isolates of *S. aureus*, *S. pyogenes*, *H. influenzae*, and *E. coli* were chosen for determining the antimicrobial properties of the samples as these organisms are known common bacterial pathogens of both humans and animals. A total of six bacterial strains were studied, two *S. aureus* strains (one was American Type Culture Collection (ATCC) strain 25,923 and the other was a clinical isolate obtained from the wound of a canine), two *E. coli* strains (ATCC strain 25,922 and a clinical isolate from the wound of a canine), one *H. influenzae* (ATCC strain 19,418) and one *S. pyogenes* (ATCC strain 19,615) were tested. All ATCC strains were obtained from a stock culture collection of the Medical Microbiology Department at the Faculty of Medical Sciences, the University of the West Indies.

### Culture conditions of the bacterial strains

Blood agar plates and chocolate agar plates were prepared using Oxoid® brand media (Basingstoke, Hampshire, England), according to the manufacturer’s instructions. *S. aureus*, *S. pyogenes* and *E. coli* were subcultured onto blood agar, to ensure purity of colonies while *H. influenzae* was subcultured onto chocolate agar. All plates were incubated aerobically at 37 °C for 24 h (CO_2_ was supplemented for *H. influenzae*). Pure cultures were then used in the broth microdilution and agar diffusion assay.

### Semi-quantitative susceptibility testing using the broth microdilution assay

The broth microdilution assay was performed in triplicate and in accordance with the protocol described by the Clinical Standards and Laboratory Institute (2015) reference method M7-A7 for bacteria [39]. Flat-bottom 96-well microtitre plates were utilized for the mixing and incubation. All wells were labelled and assigned concentrations and honey types. Mueller-Hinton broth was prepared using Oxoid® brand media (Basingstoke, Hampshire, England).

Briefly, two-fold serial dilutions of all honey samples were prepared using cation-adjusted Mueller-Hinton broth (CAMHB) (which was used as the diluent for honey being inoculated with *S. aureus* and *E. coli* isolates) and CAMHB supplemented with 5% lysed horse blood (LHB) (which was used as the diluent for honey being inoculated with *S. pyogenes* and *H. influenzae*), yielding 64, 32, 16, 8, 4 and 2% w/v honey concentrations respectively. Then, 50 μl of the diluted honey of the different concentrations were place into their respective wells. All wells were filled with 40 μl CAMHB or CAMHB+LHB (depending on the identity of the inocula). Concurrently, 0.5 McFarland standard suspensions were made by diluting colonies from each of the six isolates in sterile 0.85% saline producing suspensions containing 1 × 10^8^ CFU/mL of bacteria. These suspensions were further diluted yielding a final bacterial concentration of 5 × 10^6^ CFU/ml or 5 × 10^4^ CFU/well in a volume of 100 μl per well. The final concentrations of honey ranged from 32 to 1% respectively. The control wells contained dilutions of honey with CAMHB or CAMHB+LHB and wells with CAMHB or CAMHB+LHB with inocula and without honey.

All plates were incubated at 37 °C for 24 h (CO_2_ for *H. influenzae*), after which, 10 μl were sub-cultured from non-turbid wells onto BAP and incubated for 24 h at 37 °C. This enabled detection of the MIC (which is the lowest concentration of honey in which bacterial growth was greatly inhibited and MBC (which is the lowest concentration of honey in which the bacteria were killed).

### Agar diffusion assay

**Fig. 1:**
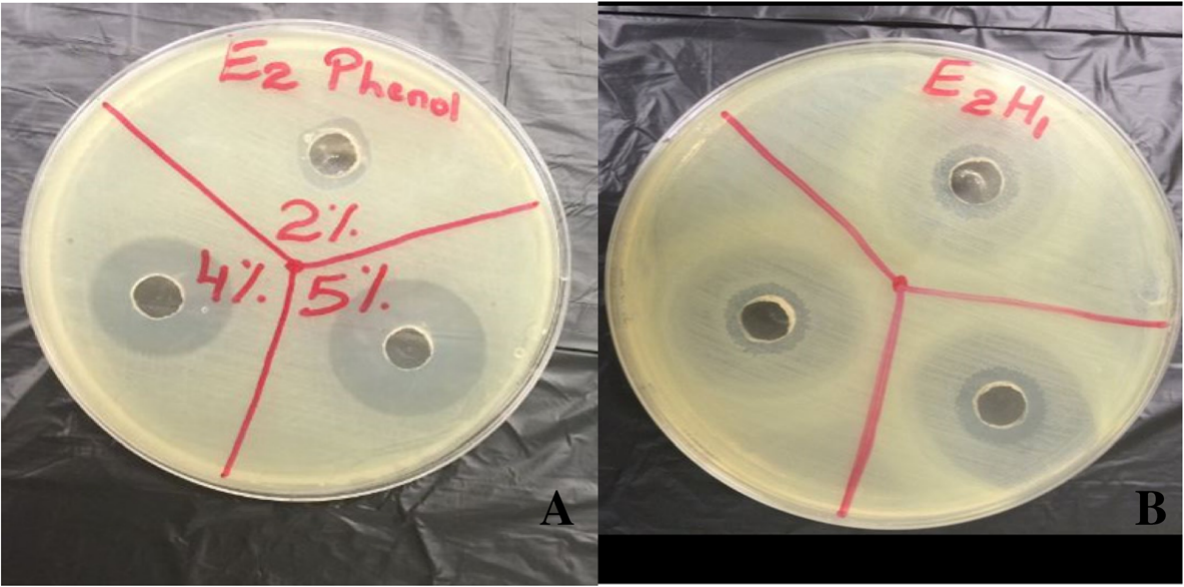
Mueller-Hinton Agar (MHA) plates divided into thirds, with wells for honey samples and phenol standards which were used in the agar diffusion assay A) Zones of inhibition produced by phenol (in the wells) against the clinical *E. coli* isolate (as the lawn) B) Zones of inhibition produced by *M. favosa* honey of Tobago (in the wells) against the clinical *E. coli* isolate (as the lawn)

This protocol was performed according to Boorn et al. 2010. A 50% w/v honey solution was prepared using sterile distilled water [12]. A 0.5 McFarland turbidity standard (1 × 10^8^ CFU/ml) suspension of each organism was made using sterile saline as the diluent. For each isolate, a Mueller-Hinton Agar (MHA) plate (or chocolate agar for *H. influenzae*) was divided into thirds (Fig. 1) and a sterile swab was used to inoculate a bacterial lawn. An 8-mm diameter well was cut into each section of agar and 100 μl of freshly prepared honey solution (less than two hours old) was added to each well. Six concentrations (2, 4, 5, 6, 8 and 10% w/v) of honey and phenol were used. Phenol was used as the standard and this assay was performed in triplicate.

The plates were incubated at 37 °C for 24 h (CO_2_ was supplemented for *H. influenzae*). The zones of inhibition were measured to the nearest millimetre (using an Antibiotic Zone Scale-C™), and the mean zone size was calculated for each honey sample. A graph of the phenol concentration against the mean zone of inhibition squared was plotted and the equation from the best-fit straight line was used to facilitate calculation of the activity of the diluted honey samples. The dilution factor was calculated and the y-values were substituted into the equation of the line to determine the x-values (phenol equivalence).

## Results

The sugar content of the honey samples ranged from 66.0 to 81.6%. The lowest sugar content was observed in fresh *F. nigra* honey from Tobago and the highest was observed in *A. mellifera* honey from Trinidad (Table 1). The moisture content, ranged from 16.9 to 32.4%, aged *A. mellifera* contained the least while the fresh *F. nigra* honey from Tobago contained the most moisture. The pH ranged from 2.88 to 3.91, lower pH values were observed in the honey samples of Tobago, with *M. favosa* honey displaying the lowest pH of all the honeys tested. The specific gravity ranged from 1.34 to 1.45. The lowest values for specific gravity were observed in fresh *F. nigra* and *A. mellifera* honeys and the highest value was observed in aged *A. mellifera* honey. The values for the artificial honey were not included in the ranges because the artificial honey was used as a control in the broth microdilution and agar diffusion assay.

**Table 1:** Descriptions and physical characteristics of the honey samples

Type of bee	Origin of the honey	Location from which honey was obtained	Age of honey	Appearance of the honeys	Sugar content (%)	Moisture content (%)	pH	Specific gravity
Stingless	*Frieseomelitta nigra*	Trinidad	Fresh		70.0	28.4	3.17	1.37
		Tobago	Fresh		66.0	32.4	3.12	1.34
	*Melipona favosa*	Tobago	Fresh		73.5	24.9	2.88	1.39
Stinging	*Apis mellifera*	Trinidad	Fresh		80.5	17.9	3.91	1.34
		Trinidad	Aged		81.6	16.9	3.33	1.45
	Artificial honey	Made at the laboratory	40.5% fructose, 33.5% glucose, 7.5% maltose 1.5% sucrose and 17% sterile distilled water. (Used as a control)		82.5	16.0	3.61	1.46

**Fig. 2:**
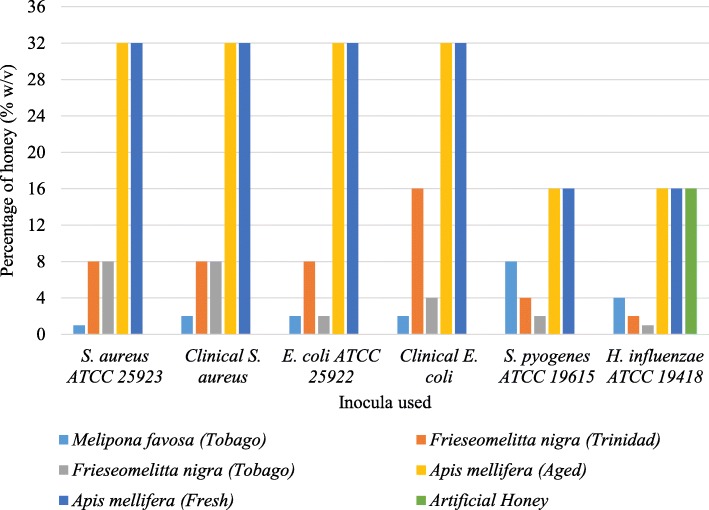
Minimum inhibitory concentrations (MICs) of the honey samples against the bacterial isolates determined by the broth microdilution test

The MIC values observed in the broth microdilution test ranged from 1 to > 32% w/v (Fig. 2). The stingless bee honeys (*F. nigra* and *M. favosa*) were inhibitory to all of the bacterial isolates utilized in this study. The MICs of the stingless bee honeys (1 to 16% w/v) were also lower than that of the *Apis mellifera* and artificial honeys (16 to > 32% w/v). Artificial honey did not display inhibitory activity against any of the isolates at the concentrations used in this study, except against *H. influenza* with a MIC of 16%.

**Fig. 3:**
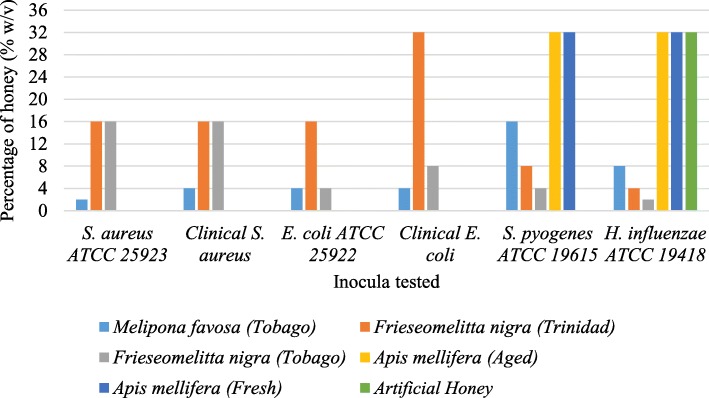
Minimum bactericidal concentrations (MBCs) of the honey samples necessary to kill the bacterial isolates as determined by the broth microdilution test. *The MBC values for aged and fresh *Apis mellifera* honey and artificial honey are not displayed for *Staph. aureus* and *E. coli* isolates because the MBC values are greater than 32%

Stingless bee honeys displayed bactericidal activity against all strains of bacteria used in this study at concentrations ranging from 2 to 16% w/v (Fig. 3). The observed MBCs were identical to or one serial dilution lower than the observed MICs, except for *Frieseomelitta nigra* honey (from Trinidad) which displayed a MIC of 16% and a MBC of 32% against the clinical isolate of *E. coli*. The stinging bee (*Apis mellifera*) honey and the artificial honey, did not display bactericidal activity against the *S. aureus* and *E. coli* strains at the concentrations used in this study. However, these honeys were bactericidal to *H. influenzae* and *S. pyogenes* at the highest concentration (32%) in this study (Fig. 3).

The zones of inhibition were measured, mean zone sizes and the phenol equivalence values were calculated and tabulated for the agar diffusion assay (Additional file 1: Tables S1-S6). Stingless bee honeys (especially those originating from Tobago), produced larger zones of inhibition than the *Apis mellifera* and artificial honey samples (Table 2). *M. favosa* of Tobago exhibited the greatest inhibitory activity against the isolates used in this study. This honey sample displayed inhibitory activity to all isolates except *S. pyogenes* ATCC strain 19,615. *M. favosa* honey of Tobago produced the largest zone sizes and phenol equivalence values observed against the *Staphylococcus* spp. isolates (28.6% w/v for the clinical isolate of *S. aureus* and 22.7% w/v for the ATCC strain of *S. aureus*) used in this study. *F. nigra* honey from Tobago exhibited the largest zones of inhibition and phenol equivalences (13.5% w/v) against *S. pyogenes*. The only inhibitory activity recorded for *A. mellifera* (stinging bee) honey, was by the fresh sample and against *S. aureus* ATCC 25923.

**Table 2:** Mean zones of inhibition in millimetres and phenol equivalence (% w/v phenol shown in brackets) as determined by the agar diffusion assay

Honey	*Staphylococcus aureus* ATCC strain 25,923	*Staphylococcus aureus* clinical isolate	*Escherichia coli* ATCC strain 25,922	*Escherichia coli* clinical isolate	*Haemophilus influenza* ATCC strain 19,418	*Streptococcus pyogenes* ATCC strain 19,615
*M. favosa* (Tobago)	27 (22.7)	27 (28.6)	12 (4.9)	14 (8.9)	11.3 (4.5)	0 (0)
*F. nigra* (Trinidad)	0 (0)	0 (0)	0 (0)	0 (0)	10.3 (3.7)	15.7 (6.5)
*F. nigra* (Tobago)	12 (4.5)	15 (8.7)	13 (5.9)	0 (0)	16 (8.1)	23 (13.5)
*A. mellifera* (Aged)	0 (0)	0 (0)	0 (0)	0 (0)	0 (0)	0 (0)
*A. mellifera* (Fresh)	10 (3.4)	0 (0)	0 (0)	0 (0)	0 (0)	0 (0)
Artificial	0 (0)	0 (0)	0 (0)	0 (0)	0 (0)	0 (0)

## Discussion

The most common type of honey available from apiaries in Trinidad and Tobago is that of the stinging bee (*Apis mellifera*). Apiaries rear only two (*Melipona favosa* and *Frieseomelitta nigra*) of the eight local stingless bee species. The volume of honey produced by stingless bees is lower than that of *Apis mellifera*. The remaining six species of native bees are seldom reared at apiaries and exist in the forests on the islands of Trinidad and Tobago. Additionally, a comparison of the local honeys with medicinal Manuka honey was not possible because pure Manuka honey is not available in Trinidad and Tobago due to restrictions on the importation of pure honey (for both medicinal and human consumption).

All of the honey samples analysed in this study were acidic with pH values for stingless bee honeys lower than that of the stinging bee and artificial honey. Fresh *M. favosa* honey displayed the lowest pH of 2.88. All stingless bee honeys evaluated in this study did not conform to the standard limits for the pH of honey (pH 3.40 to 6.1) as stated in the Codex Alimetarius [40]. These pH values were also lower than those described by Nweze et al. (2017) [41] in which an average pH of 4.21 ± 0.37 was detected for *Melipona* spp. (stingless bees) and 4.24 ± 0.20 for *Apis mellifera* from Nsukka, Nigeria. The variations between studies can be attributed to differences in geographical location, climate, botanical source, maturity of hive, harvest season and soil type, all of which influence the concentrations of the various acids present in honey [23–26].

Only fresh stingless bee honeys were available for this study, thus the effect of age was not determined. For *Apis mellifera* honey, pH values varied with age, the aged honey displayed a slightly lower pH (3.33) when compared to the fresh honey (pH 3.91). However, on comparing the MICs and MBCs of the fresh and aged honeys of the *Apis mellifera,* both honeys produced similar results, indicating that the acidity of the *Apis mellifera* honey may not be the only factor responsible for its antimicrobial activity. Other factors such as osmotic activity (due to its high sugar content), hydrogen peroxide, MGO and bee defensin-1, may be responsible.

The stingless bee honeys of Tobago (*Melipona favosa* and *Frieseomelitta nigra*) were noted to display the lowest pH, MIC and MBC values, hence for these honeys it may be possible that their antimicrobial activities may be influenced by acidity in combination with other factors. The climatic and geographical variations between Trinidad and Tobago may have also influenced the antimicrobial activities of the honey, resulting in honey from Tobago having a greater antimicrobial activity than honey of Trinidad.

The Codex Alimentarius standards for honey marketed as food, indicate that honey must contain no more than 20% moisture and no less than 60% sugar. The stingless bee honeys used in this study all exceeded this standard as they contained greater that 20% moisture (24.9 to 32.4%) while the *Apis mellifera* honey was within range (16.9% for aged and 17.9% for fresh). These findings suggest that the antimicrobial activity of the honey may be influenced over time indicating a finite shelf-life for this product. Higher moisture content of honey results in an increased possibility for the growth of yeast which causes fermentation of the honey and spoilage.

A study published by Bijlsma et al. in 2006 [35], also detected higher than 20% moisture contents for various species of stingless bees in Trinidad and Tobago (42% for *Plebeia tobagoensis*, 36.2% for *Frieseomelitta nigra* (*Trigona)*, 31.2% for *Melipona favosa* and 32.2% for *Melipona trinitatus*). This study is in agreement with previous reports as the moisture content for *Frieseomelitta nigra* honey was 28.4 to 32.4% and for *Melipona favosa* was 24.9%.

All of the honeys used in this study contained greater than 60% sugar content which is within the limit stated in the Codex Alimentarius. The stinging bee honeys displayed sugar contents of 80.5 and 81.6% for fresh and aged honey respectively. These values were higher than that described by Nweze et al. (2017) [41] for the *Apis mellifera* in Nsukka, Nigeria, where the mean sugar content was 72.7 ± 7.5%. However, the stingless be honeys of Trinidad contained less sugar (66 to 70%) than those of the Nigerian study. These variations are due to the factors previously discussed for acidity and moisture content. The sugar content of honey is important since the higher the sugar the content the greater the osmotic pressure on the bacterial organisms.

Data obtained from the agar diffusion and phenol equivalence determinations from the standard phenol curves indicate that the stingless bee honeys originating from Tobago produced the largest zone sizes and phenol equivalence values, with *Melipona favosa* honey from Tobago showing the highest values of all the honeys against all the bacterial isolates used. This indicates that the *Melipona favosa* honey from Tobago has a broad spectrum of activity and can be used to treat infections caused by all the isolates used in this experiment. The values for the phenol equivalences for this honey ranged from 4.5% against *H. influenzae* to 22.7 and 28.6% against *S. aureus* strains. The stingless bee honeys in this study displayed slightly higher phenol equivalence values than that of *Trigona carbonaria* stingless bee honey of Australia, reported by Boorne et al. (2010) [12] in which phenol equivalences of 9.7 to 23.3% against *S. aureus* were recorded.

The phenol equivalence is an important consideration because the Unique Manuka Factor (UMF), which is a standard used to determine the potency of the Manuka honey, is based on the calculation of the phenol equivalence of the Manuka honey against *S. aureus*. Manuka honey is graded according to the UMF. Honey with a UMF less than 10 is not recommended for therapeutic use, those with a UMF of 10–15 are useful therapeutically and those with a UMF of 16–30 are highly potent with superior activity [9]. In this study phenol equivalences were obtained for the honeys against two strains of *S. aureus*. Considering this, it can be deduced that the *Melipona favosa* honey from Tobago may be comparable in activity to the Manuka honey because its phenol equivalences were more than ten. However, we have not performed chemical analyses to determine the quantities and components which are responsible for the antimicrobial activities of these local honeys.

There are many disadvantages to consider when using the agar diffusion and phenol equivalence determination method. Firstly, the strains of *S. aureus* varied between studies and are not standard. Secondly, it does not distinguish between non-peroxide and hydrogen peroxide dependent antimicrobial activity [27]. Thirdly, it is insensitive and may not detect low antibacterial activity and finally, there may be great variability in the results due to variations in the volume of agar used, concentration of inocula and incubation period [12]. Another point to note is that in vitro activities reported here may not be reflective of the in vivo activity, for example staphylococci produce and reside in biofilms which may offer some protection against effects of antimicrobial agents.

Although it is not possible to make sound statistical deductions between the results of the broth microdilution test (MIC and MBC values) to agar diffusion test (zones of inhibition and phenol equivalences), the characteristics of the honeys used in this study are consistent between tests. Stingless bee honeys displayed greater antimicrobial activity than the stinging bee and artificial honeys. The *M. favosa* stingless bee honey from Tobago displayed a broad spectrum of activity and the greatest antimicrobial activity for both tests followed by that of *Frieseomelitta nigra* honey from Tobago.

Stingless bee honeys of *M. favosa* and *F. nigra* from Tobago displayed greater inhibitory and bactericidal activities than that of stinging bee (*A. mellifera*) and artificial honey (control), on the isolates used in this experiment. Hence these stingless bee honeys from Tobago are of potential medicinal value. However, these honeys do not conform to the values stipulated by the Codex Alimentarius on pH and moisture content which may be prohibitive in allowing them to be commercially marketed as honey as food. Additionally, local farmers cannot afford the numerous tests required for certification as medicinal honey for marketing and distribution on a global scale.

## Conclusion

This is the first investigation of local honeys to determine the physical and chemical characteristics of honey samples (specific gravity, pH and sugar and moisture content). MICs and MBCs were determined using the broth microdilution technique and phenol equivalence values were calculated using the agar diffusion technique. These tests yielded positive results by demonstrating the antimicrobial activity of honey from Trinidad and Tobago. Due to limited resources a comprehensive study inclusive of melissopalynological analyses and other chemical analyses to determine the concentration of hydrogen peroxide, MGO bee defensin-1 and other chemicals with potential antimicrobial activity were beyond the scope of this study. This data, though preliminary, will contribute to the global understanding of the medicinal potential of honeys from Trinidad and Tobago and aid in gaining support for further research in this area.

## Supplementary information

**Additional file 1: Tables S1-S6.** The phenol equivalence values calculated for each isolate used and the mean zone sizes produced by each honey. File name: Phenol equivalence supplementary data.xlsx.

## Data Availability

Data are available from the corresponding author upon reasonable request and the approval of the data owner.

## Abbreviations

ATCC: American *Type Culture Collection*
CAMHB: Cation-adjusted Mueller-Hinton Broth
*E. coli*: *Escherichia coli*
*H. influenzae*: *Haemophilus influenzae*
LHB: Lysed horse blood
MBC: Minimum Bactericidal Concentration
MIC: Minimum Inhibitory Concentration
*S. aureus*: *Staphylococcus aureus*
*S. pyogenes*: *Streptococcus pyogenes*
